# Diabetes and gut microbiome

**DOI:** 10.3389/fmicb.2024.1451054

**Published:** 2025-01-07

**Authors:** Kateřina Olša Fliegerová, Tiziana Maria Mahayri, Hana Sechovcová, Chahrazed Mekadim, Jakub Mrázek, Radka Jarošíková, Michal Dubský, Vladimíra Fejfarová

**Affiliations:** ^1^Laboratory of Anaerobic Microbiology, Institute of Animal Physiology and Genetics, CAS, Prague, Czechia; ^2^Department of Veterinary Medicine, University of Sassari, Sassari, Italy; ^3^Department of Microbiology, Nutrition and Dietetics, Faculty of Agrobiology, Food and Natural Resources, Czech University of Life Sciences, Prague, Czechia; ^4^Institute for Clinical and Experimental Medicine, Diabetes Centre, Prague, Czechia; ^5^Department of Internal Medicine, Second Faculty of Medicine, Charles University, Prague, Czechia

**Keywords:** gut microbiota, diabetes mellitus, T1DM, T2DM, antidiabetic drugs

## Abstract

Diabetes mellitus represents a significant global health problem. The number of people suffering from this metabolic disease is constantly rising and although the incidence is heterogeneous depending on region, country, economic situation, lifestyle, diet and level of medical care, it is increasing worldwide, especially among youths and children, mainly due to lifestyle and environmental changes. The pathogenesis of the two most common subtypes of diabetes mellitus, type 1 (T1DM) and type 2 (T2DM), is substantially different, so each form is characterized by a different causation, etiology, pathophysiology, presentation, and treatment. Research in recent decades increasingly indicates the potential role of the gut microbiome in the initiation, development, and progression of this disease. Intestinal microbes and their fermentation products have an important impact on host metabolism, immune system, nutrient digestion and absorption, gut barrier integrity and protection against pathogens. This review summarizes the current evidence on the changes in gut microbial populations in both types of diabetes mellitus. Attention is focused on changes in the abundance of specific bacterial groups at different taxonomic levels in humans, and microbiome shift is also assessed in relation to geographic location, age, diet and antidiabetic drug. The causal relationship between gut bacteria and diabetes is still unclear, and future studies applying new methodological approaches to a broader range of microorganisms inhabiting the digestive tract are urgently needed. This would not only provide a better understanding of the role of the gut microbiome in this metabolic disease, but also the use of beneficial bacterial species in the form of probiotics for the treatment of diabetes.

## 1 Introduction

Diabetes mellitus (DM) is a metabolic disease characterized by hyperglycemia resulting from insufficient control of blood glucose levels and impaired insulin secretion (Petersmann et al., 2018). DM is classified into several categories. The main types are type 1 (T1DM) and type 2 (T2DM), and other forms include maturity-onset diabetes of the young (MODY) (Horikawa, 2018), gestational diabetes (Buchanan and Xiang, 2005), neonatal diabetes (Beltrand et al., 2020), steroid-induced diabetes or Latent Autoimmune Diabetes in Adults (LADA) (Carlsson, 2019) and genetically determined metabolic disorders connected with abnormal glucose metabolism (Vekic et al., 2022). Diabetes prevalence in Europe is very high. According to the International Diabetes Federation, 9.2% of people suffer from DM and this number is expected to increase to 13% by 2045 (International Diabetes Federation, 2021). China and India are particularly affected by this disease, experiencing a dramatic increase in T2DM prevalence despite having a relatively low rate of obesity (DeFronzo et al., 2015). The reasons for the development of diabetes can be different and depend on many aspects. Genetic predisposition, family history of diabetes, health status, ethnic background, inactive lifestyle, unhealthy eating habits, and obesity are considered the most common risk factors that can lead to diabetes (Gowd et al., 2019; Li W.-Z. et al., 2020). The rapid increase in disease incidence, which is expected to reach 700 million people worldwide by 2045 (Saeedi et al., 2019), indicates that environmental factors presumably play a key role in this phenomenon. In the last decade, the role of the gut microbiota in the development of this disease has been implicated as an important factor. Based on the knowledge that human intestinal microbiome play an essential role in health and disease, including the development of a fully functional immune system (Lynch and Pedersen, 2016; Zheng et al., 2020), the gut microbiome received considerable attention as a phenomenon involved in the development and progression of DM disease. The causal relationship between gut bacteria and diabetes is still unclear, and future studies on the pathophysiological role of the gut microbiome in DM are expected and needed. The current status of research and understanding of the relationship between gut microorganisms and diabetes is described here, with a focus on the human microbiome.

## 2 Pathogenesis of type 1 and type 2 DM

T1DM and T2DM are the most common subtypes. Type 1 occurs mainly in children or adolescents (Katsarou et al., 2017), while type 2 usually affects middle-aged and elderly adults who have persistent hyperglycemia mainly due to genetic variants, inappropriate lifestyle and dietary habits. The pathogenesis of these two types is meaningly different, so each type is characterized by a different etiology, pathophysiology, presentation, and treatment.

T1DM is characterized by elevated blood glucose levels (hyperglycemia) caused by deficient insulin production due to destruction of the *β*-cells of the pancreatic islets of Langerhans predominantly as a result of autoimmune inflammation (Katsarou et al., 2017). The classic view is that autoreactive T cells mistakenly destroy healthy *β*-cells, resulting in insulin deficiency that causes hyperglycemia in patients with subsequent overproduction of glucose by the liver via glycogenolysis and gluconeogenesis enhanced by glucagon and decreased ability of glucose cellular uptake and degradation by peripheral tissues, such as muscle and adipose tissue (Eiselein et al., 2004). However, recent opinions suggest the alternative view that the key contributors to disease are the *β*-cells themselves, which dysfunction and destruction is controlled by their own metabolic activity. In fact, β-cells are susceptible to biosynthetic stress and have limited self-defense mechanisms. When under stress, β-cells trigger an immune response that can significantly impair the production of a vital hormone (Roep et al., 2020).

While patients with T1DM are rarely obese (but the presence of obesity is not incompatible with the diagnosis), T2DM is closely associated with obesity and metabolic syndrome. Type 2 diabetes is a metabolic disease in which the driving force for insulin resistance and impaired insulin secretion lies in patient’s overweight or obesity. Obesity is characterized by chronic low-grade inflammation associated with increased secretion of pro-inflammatory cytokines from adipose tissue and infiltration of leukocytes, including macrophages, into adipose tissue. Chronic inflammation thus impairs insulin signaling in adipocytes, leading to insulin resistance and the development of metabolic disorders (Kim and Nam, 2020). Approximately 86% of T2DM patients are overweight (Thingholm et al., 2019) and obesity-induced insulin resistance is the major underlying pathophysiological factor. T2DM is more common than T1DM and accounts for more than 90% of all cases. These data indicate that this disease has become a global pandemic and the number of patients is rapidly increasing, especially in industrialized countries (Khan et al., 2020).

DM patients suffer from acute and chronic complications. Hypoglycemia and hyperglycemia are the most frequent acute symptoms and in certain cases could be life-threatening. Chronic complications are common in both T1DM and T2DM patients and are responsible for significant morbidity and mortality. These types of complications are generally divided into microvascular and macrovascular. Microvascular complications are more common and include neuropathy, diabetic kidney disease, and retinopathy. Macrovascular complications induce cardiovascular comorbidities leading to ischemic heart disease, stroke and peripheral artery disease (Lotfy et al., 2017). Diabetic foot (DF) is also a common problem associated with diabetes, which can ultimately lead to lower limb amputation (Tuttolomondo et al., 2015; Yazdanpanah et al., 2018; Pourkazemi et al., 2020 Sechovcová et al., 2023). Growing evidence also indicate that lung injury could be one of the long-term consequences of diabetes (Wang G. et al., 2021).

## 3 Healthy intestinal microbiome

Microorganisms in the gastrointestinal tract have received considerable attention in the last two decades, and intensive research on the gut microbiota and microbiome has revealed their irreplaceable importance for human health. Intestinal bacteria, together with archaea, fungi, and viruses, are now considered a “virtual organ” providing essential metabolic functions which cannot be performed by the mammalian host (Koboziev et al., 2014). Apart from the obvious contribution to degradation of complex carbohydrates, resulting in the production of energy and health important short-chain fatty acids (SCFA), there are many other critical functions that have been gradually discovered. Gut microbiota synthesizes essential vitamins, eliminates toxins, maintains the integrity of the intestinal epithelium, regulates development, homeostasis, and function of the innate and adaptive immune systems, regulates gut endocrine function and neurological signaling, produces neuroactive compounds, and participates in a bidirectional network of signaling pathways known as the gut-brain axis (Morowitz et al., 2011; Koboziev et al., 2014; Rutsch et al., 2020; Zheng et al., 2020; Fan and Pedersen, 2021) and gut-skin axis (De Pessemier et al., 2021; Patel et al., 2022). Recent research also shows a close correlation between the gut microbiome and respiratory disorders referred to as a gut-lung axis (Enaud et al., 2020; Tang et al., 2021).

Human gut microbiota is rich consortium of microorganisms, in which bacteria form the most numerous part, reaching a density of about 10^12^ cells/g of colon contents. About 500–1,000 different species are highly variable at lower taxonomic levels, but generally belong to four dominant phyla. Firmicutes and Bacteroidetes account for more than 90% of the population, while Actinobacteria and Proteobacteria are less represented (< 1–5%) (Almeida et al., 2019; Rutsch et al., 2020). Other phyla, including Verrucomicrobia, Planctomycetes, and Spirochaetes, have very low abundance but contain some important beneficial bacterial species (e.g., *Akkermansia muciniphila* in Verrucomicrobia) (Rinninella et al., 2019). Despite their low abundance, members of the rare biosphere can play a crucial role in the gut microbiome’s metabolic processes. They may significantly enhance microbial diversity and contribute to the stability of the human gut. Intestinal rare taxa may be involved in detoxification of ingested chemicals, digestion of unusual food components and stimulation of the human immune system, thus promoting overall gut health and resilience (American Society for Microbiology, 2011; Bhute et al., 2017a).However, no uniform optimal composition of intestinal microbiome can be determined because the gut microbiota of each individual is different, highly variable at the genus level, and characterized by a specific combination of bacterial species (Rinninella et al., 2019). Therefore, the concept of a healthy microbiome cannot be described unambiguously. On the other hand, there are some generally accepted parameters, characteristics and taxonomic traits associated with a healthy gut. Species richness, diversity, and a stable functional microbial core are indicative of a good gut condition. The richer and more diverse the microbiome, the better the intestinal community resists extrinsic threatening influences (Rinninella et al., 2019; Manor et al., 2020). This is associated with the high functional response diversity, which is defined as the extent to which species in a community that contribute to the same ecosystem function vary in their sensitivity to changes in the ecosystem (Lozupone et al., 2012). Research has shown that a richer, more diverse and balanced microbiome composition, referred to as eubiosis (Iebba et al., 2016; Bajinka et al., 2020), represents a promising therapeutic target for addressing various conditions influenced by the microbiome, including infections caused by serious pathogens (Goldberg et al., 2014; Seekatz et al., 2022). Another prerequisite for a healthy microbiome is the presence of bacterial species capable of decomposing structural polysaccharides in the diet. This fermentation results in the production of short-chain fatty acids (SCFAs), and important is also the biosynthesis of some essential amino acids and vitamins. SCFAs, mainly acetate, butyrate, and propionate, have a positive effect on intestinal health by maintaining intestinal barrier integrity, mucus production, protection against inflammation, glucose homeostasis, and immunomodulation (Silva et al., 2020; Portincasa et al., 2022). SCFAs influence the occurrence and development of various diseases, and the roles of individual volatile fatty acids vary depending on the disease (Fan and Pedersen, 2021; Zhang D. et al., 2023). Of particular importance is butyrate, which provides an energy source for colonocytes and plays a significant physiological role (Singh et al., 2023). Major butyrate producers in the human gut include strains of *Faecalibacterium prausnitzii, Eubacterium rectale, Eubacterium hallii, Ruminococcus bromii*, *Butyricicoccus pullicaecorum*, *Roseburia* spp. (*R. faecis*, *R. inulinivorans*, *R. intestinalis*, *R. hominis*) and *Anaerostipes* spp. (*A. butyraticus, A. caccae*, *A. hadrus*) (Louis et al., 2010; Plöger et al., 2012; Rivière et al., 2016) (see Table 1). King et al. (2019) recently published a list of healthy human reference microbiome and an abundance profile. This list, called GutFeelingKB, is based on the combination of the complete National Center for Biotechnology Information NCBI non-redundant nucleotide database and sequences of healthy people from the Human Microbiome Project (HMP). A comprehensive methodology was used to link bacterial species with intestinal health. Healthy participants provided fecal samples, which were sequenced and analyzed using bioinformatic pipelines. CensuScope and HIVE-hexagon were used to generate taxonomic profiles, while MaAsLin and cosine similarity coefficients linked bacterial abundance to clinical and dietary data. The resulting profile provides a summary of 157 organisms (8 phyla, 18 classes, 23 orders, 38 families, 59 genera, and 109 species) that can be used as healthy controls for studies related to dysbiosis.

**Table 1:** Metabolic products and function of beneficial gut bacteria.

Bacteria	Metabolic products	Function	References
*Faecalibacterium prausnitzii*	Production of butyrate, D-lactate, formate, bioactive anti-inflammatory molecules such as shikimic and salicylic acids; utilization of acetate	Anti-inflammatory effects	Ferreira-Halder et al. (2017) and Parsaei et al. (2021)
*Eubacterium rectale*	Production of butyrate, lactate, acetate, occasionally propionate and succinate	Anti-inflammatory effects	Cockburn et al. (2015) and Lu et al. (2022)
*Eubacterium hallii*	Production of acetate, formate, butyrate	Anti-inflammatory effects and prevention from lactate accumulation	Duncan et al. (2004) and Mojgani and Dadar (2021)
*Ruminococcus bromii*	Production of acetate, butyrate, formate, acetate and ethanol	Production of energy from resistant starch	Ze et al. (2015), Lal et al. (2022), and Peterson et al. (2022)
*Butyricicoccus pullicaecorum*	Production of butyrate	Regulation of short-chain fatty acid transporter and receptor, anti-inflammatory effects	Eeckhaut et al. (2013) and Chang et al. (2020)
*Roseburia faecis*	Production of butyrate, acetate; utilization of acetate	Prevention of colonic microinflammation	Tamanai-Shacoori et al. (2017) and Choi et al. (2023)
*Roseburia inulinivorans*	Production of butyrate, propionate and propanol	Anti-inflammatory effects	Scott et al. (2006) and Tamanai-Shacoori et al. (2017)
*Roseburia intestinalis*	Production of butyrate, formate, lactate; utilization of acetate	Prevention of intestinal inflammation and maintenance of energy homeostasis	Tamanai-Shacoori et al. (2017) and Nie et al. (2021)
*Roseburia hominis*	Production of butyrate; utilization of acetate	Prevention of intestinal inflammation and immune maintenance	Tilg and Moschen (2014) and Tamanai-Shacoori et al. (2017)
*Anaerostipes butyraticus*	Production of butyrate; utilization of acetate and propionate	Gut health beneficial	Eeckhaut et al. (2010)
*Anaerostipes caccae*	Production of butyrate, acetate, lactate; utilization of acetate	Regulating food allergies in early life	Feehley et al. (2019) and Chia et al. (2020)
*Anaerostipes hadrus*	Production of formate, butyrate; utilization of D-lactate and acetate	Gut health beneficial	Allen-Vercoe et al. (2012) and Kant et al. (2015)
*Bifidobacteria*	Production of acetate, lactate, formate; no production of butyrate	Role in gut homeostasis and stimulation of immune system, improve general health and reduce disease risk, the most common probiotic	Arboleya et al. (2016) and Usta-Gorgun and Yilmaz-Ersan (2020)
*Lactobacilli*	Production of lactate, acetate, ethanol; no production of butyrate	Maintenance of intestinal barrier integrity, protection from infections, regulation of the immune system, the most common probiotic	Dempsey and Corr (2022) and Huang et al. (2022)
*Akkermansia muciniphila*	Production of acetate, propionate, butyrate, branched-chain fatty acids (isobutyric and isovaleric acids)	Maintenance of intestinal mucus integrity, reduction of intestinal permeability, anti-inflammatory and immunomodulatory effects, improving of insulin sensitivity	Li et al. (2021, 2022) and Rodrigues et al. (2022)

Lower microbiome diversity and low abundance of SCFA-producing bacteria is a common feature of many diseases, not only chronic intestinal inflammations such as Crohn’s disease and ulcerative colitis, but also most metabolic and autoimmune diseases, including both types of diabetes mellitus.

A recent article of Wu et al. (2024) also makes a remarkable advance in the definition of a healthy microbiome. The authors constructed co-abundance networks of high-quality metagenome-assembled genomes based on 26 datasets including healthy controls, individuals under various environmental perturbations, dietary regimes and patients with 15 different diseases and discovered a core set of health-relevant microbiomes. All bacteria from the beneficial microbiome of the group of healthy subjects belonged to the phylum Firmicutes. Their genomes were rich in CAZy genes, which are crucial for the dietary fiber digestion (cellulose, arabinoxylan) and the production of butyrate, but had a lower proportion of genes for inulin utilization and propionate production. The detrimental microbiome was composed of five different phyla, including Firmicutes, Bacteroidota, Proteobacteria, Actinobacteriota, and Fusobacteriota, and their genomes were rich in genes conferring antibiotic resistance and expressing virulence factors that may activate a pathogenic interaction with the host. Surprisingly, the data indicate that a high abundance of certain species of microbes is not so important as previously thought, while stable microbial interaction is critical parameter of healthy microbiome (Wu et al., 2024).

## 4 Gut microbiota and T1DM

The role of the gut microbiome in T1DM activation is poorly understood, and there is still insufficient evidence to unequivocally support the idea that intestinal microorganisms activate this disease. A causal relationship between the gut microbiota and T1DM has not yet been disclosed and remains unclear. The complexity and diversity of the microbiota, as well as inter-individual variations, make it difficult to confirm cause-effect relationships. In addition, the interactions between microbiota and disease can be bidirectional, making it even more difficult to provide clear evidence. Therefore, it is not yet evident whether gut microbial changes are the cause or the effect of the disease. Understanding this relationship is further complicated by the fact that the onset and manifestation of the disease occurs in children and young adults, whose intestinal microbiota undergoes dynamic development (Knip and Siljander, 2016). However, the link between the pathogenesis of insulin dysfunction and alterations in the microbial composition of people suffering from T1DM seems obvious. Several studies have observed reduced bacterial diversity and low numbers of butyrate-producing bacteria (Brown et al., 2011; Giongo et al., 2011; Vaarala, 2012; De Goffau et al., 2013; Qi et al., 2016). Analysis at the family level indicated in T1DM subjects significantly increased *Bacteroidaceae* (De Goffau et al., 2013; Leiva-Gea et al., 2018), *Rikenellaceae* (Giongo et al., 2011; Leiva-Gea et al., 2018), *Prevotellaceae* (Leiva-Gea et al., 2018), *Ruminococcaceae* (Leiva-Gea et al., 2018), *Veillonellaceae* (Giongo et al., 2011; Leiva-Gea et al., 2018), *Streptococcaceae* (Leiva-Gea et al., 2018), and *Enterobacteriaceae* (Soyucen et al., 2014; Leiva-Gea et al., 2018), while *Lachnospiraceae* (Giongo et al., 2011; Leiva-Gea et al., 2018), *Bifidobacteriaceae* (Leiva-Gea et al., 2018) and *Eubacteriaceae* (Giongo et al., 2011) were significantly more abundant in healthy controls. At the genus level the T1DM subjects were enriched with *Bacteroides* (Brown et al., 2011; Giongo et al., 2011; De Goffau et al., 2013; Leiva-Gea et al., 2018), *Prevotella* (Leiva-Gea et al., 2018), *Blautia* (Qi et al., 2016; Leiva-Gea et al., 2018), *Veillonella* (Brown et al., 2011; Leiva-Gea et al., 2018), *Streptococcus* (Brown et al., 2011; De Goffau et al., 2014; Leiva-Gea et al., 2018), *Clostridium* (Giongo et al., 2011; De Goffau et al., 2013, 2014), *Sutterella* (Leiva-Gea et al., 2018), *Enterobacter* (Leiva-Gea et al., 2018), *Alistipes* (Brown et al., 2011), and *Ruminococcus* (Giongo et al., 2011; De Goffau et al., 2014; Leiva-Gea et al., 2018).

On the other hand, the abundance of strains of *Lachnospira* (Qi et al., 2016; Leiva-Gea et al., 2018), *Roseburia* (Brown et al., 2011; De Goffau et al., 2013; Cinek et al., 2018; Leiva-Gea et al., 2018), *Anaerostipes* (Brown et al., 2011; Leiva-Gea et al., 2018), *Faecalibacterium* (Brown et al., 2011; Giongo et al., 2011; Huang et al., 2018; Leiva-Gea et al., 2018), *Eubacterium* (Brown et al., 2011; Giongo et al., 2011; Cinek et al., 2018), *Bifidobacterium* (Soyucen et al., 2014; Leiva-Gea et al., 2018), *Akkermansia* (Brown et al., 2011), and *Lactobacillus* (De Goffau et al., 2014; Alkanani et al., 2015) was reduced in diabetics with T1DM and significantly increased in healthy controls. However, in the work of Brown et al. (2011), *Prevotella* was much more abundant in controls, and the lactate producers including *Lactobacillus*, *Lactococcus*, and *Bifidobacterium* were increased in T1DM cases, contradicting the findings of Leiva-Gea et al. (2018) and Alkanani et al. (2015). Several strains of *Bifidobacteria* and *Lactobacilli* have been nevertheless successfully tested as probiotics showing beneficial effects both in pediatric (Groele et al., 2017; Kumar et al., 2021) and adult (Ahola et al., 2017; Zhang X. et al., 2023) T1DM patients. However, the effect strongly depends on the particular strain(s) and it still remains questionable which probiotic bacteria are potentially the most useful for treatment of T1DM (Groele et al., 2021).

It is evident that the aforementioned shifts in the microbiome of T1DM individuals have been observed in a number of studies and seem to be characteristic for diabetes 1 disease, but on the other hand, the results are highly dependent on geographic location, age, gender, and diet. Cinek et al. (2018), who studied children and adolescents from African and Asian countries, described a positive association of T1DM with the genus *Escherichia*, which was twice more abundant in patients compared to controls. Huang et al. (2018) observed increased *Ruminococcaceae* and *Veillonellaceae* in healthy subjects in a Chinese cohort, which contradicts the findings of Leiva-Gea et al. (2018) and Giongo et al. (2011). In studies with non-European populations (Mejía-León et al., 2014; Cinek et al., 2018; Huang et al., 2018), no decreased bacterial diversity was observed in T1DM patients. Alkanani et al. (2015) and De Goffau et al. (2013, 2014) indicated that changes in abundances of certain bacterial groups, particularly in the Bacteroides phylum, may be age-dependent, in facts, Bacteroidetes were found to be more prevalent in the younger diabetic children and this finding was also observed in the recent work of Mokhtari et al. (2021). In particular, the genus Bacteroides, seems to play a crucial role in the development of T1DM, probably through the production of glutamate decarboxylase, which can induce glutamic acid decarboxylase autoimmunity via molecular mimicry (De Goffau et al., 2014). Giongo et al. (2011) observed the decreased diversity with increasing age in young children with development of T1DM. Bacterial changes associated with diabetes are summarized in Supplementary Table S1 showing unambiguous findings, while Supplementary Table S2 outlines controversial results achieved by different author groups.

T1DM is a complex disease that affects a variety of biochemical and immunological processes in the body, and research brings many other important new findings. One interesting aspect is changes in the metabolomic profile, which reflect the autoimmune nature of diabetes. Lipidomic changes are characterized by higher levels of low-carbon-number saturated lipid classes, including myristic, stearic and palmitic acid (Arneth et al., 2019), and lower levels of certain sphingolipids and glycerophospholipids (Greenhill, 2018; Arneth et al., 2019). The shift in amino acids (AA) profile is often associated with an increase in circulating AA, particularly branched chain AA, during insulin deprivation in T1DM individuals (Charlton and Nair, 1998; Hebert and Nair, 2009). Research also shows that T1DM patients suffer from altered intestinal barrier function (Dedrick et al., 2020). The increased gut permeability, often referred to as leaking gut syndrome, allows antigens and pathogens to cross the intestinal barrier, which may trigger or exacerbate immune responses against pancreatic beta cells, contributing to the autoimmune attack characteristic for T1DM (Vaarala, 2018; Li and Atkinson, 2015). This phenomenon once again emphasizes the significance and importance of the intestinal microorganisms. Microbiota-host interactions significantly influence immune regulation and inflammatory responses, which are central to the autoimmune destruction of pancreatic beta cells (Majumdar et al., 2022). Dysbiosis in T1DM is associated with an increase in pro-inflammatory cytokines, such as IL-17 and IFN-*γ*, which can further exacerbate the autoimmune attack on pancreatic tissue (Li et al., 2015; Roep et al., 2020). Changes in microbial composition can therefore modulate immune signaling and influence the progression of T1DM.

Diet and lifestyle definitely have a significant impact on onset and incidence of T1DM, and both aspects are closely related to the composition of the gut microbiome. In childhood, breastfeeding and late inclusion of gluten, fruit, and cow’s milk in the diet may reduce the risk of T1DM (Lampousi et al., 2021). On the other hand, over the life course, increased risk is associated with inappropriate dietary habits, such as frequent consumption of foods and beverages with a high glycemic index (GI) and a diet low in fiber and rich in saturated fats (Piłaciński and Zozulińska-Ziółkiewicz, 2014).

All of these aspects and factors are reflected in the varying geographic distribution of T1DM, with lower incidence in most Asian populations (with the exception of Kuwait) and low or intermediate incidence in African populations. Incidence in South American populations varied from very low to high, and the highest incidence was found in North American and European populations. Finland, Sweden, and Norway are known to be the three countries with the highest T1DM incidences (Paun et al., 2017; Xia et al., 2019). However, the incidence of this disease is on the rise worldwide, especially among children. Regions that previously had a lower incidence tend to show a steep annual increase, whereas countries with an already established high incidence show a moderate increase or even stabilization in the incidence of T1DM (Xia et al., 2019). Geographic location is undoubtedly associated with a specific type of diet that influences the gut microbiome. Exploring this relationship certainly deserves attention and could help clarify the link between dietary habits and T1DM. A proinflammatory diet (mainly rich in high-fat dairy products and sugar-sweetened beverages and low in fiber content and vegetable) can induce alterations in the composition of the microbiota and may lead to intestinal inflammation and modification of the metabolic and immunological profile within the intestinal mucosa of patients with TD1M (Vatanen et al., 2018). Notably, the onset of T1DM is usually preceded by clinical signs of modification on mucus layer and intestinal inflammation associated with lymphocyte infiltration, increased permeability, and the presence of inflammatory cytokines within the intestinal mucosa (Secondulfo et al., 2004; Sorini et al., 2019). The functional impairment of gut barrier integrity is linked to the pathogenesis of T1DM rather than being a secondary effect of diabetes-induced metabolic changes. It can lead to the uncontrolled passage of bacterial components into the systemic circulation, directly contributing to beta cell damage, or activating beta cell autoimmunity through pancreatic lymph nodes and tissues (Sun et al., 2015). An alternative mechanism involves the activation of autoimmune T cells specifically targeting beta cell antigens. These T cells are activated by the presentation of the cognate antigens by professional antigen-presenting cells in an inflammatory context. Research emphasizes the critical role of the intestinal innate immune system in disrupting T cell tolerance to islet beta cells during episodes of gut dysbiosis. The changes in gut microbial diversity can cause a shift toward pro-inflammatory state, which compromises the integrity of the intestinal barrier. This weakened barrier allows bacterial components to penetrate the leaky gut and trigger activation of innate immune cells. The resulting inflammatory state creates favorable conditions for the activation of diabetogenic T cells, which contribute to beta cell damage, ultimately leading to the development of autoimmune type 1 diabetes (Turley et al., 2005; Majumdar et al., 2022).

## 5 Gut microbiota and T2DM

**Figure 1:**
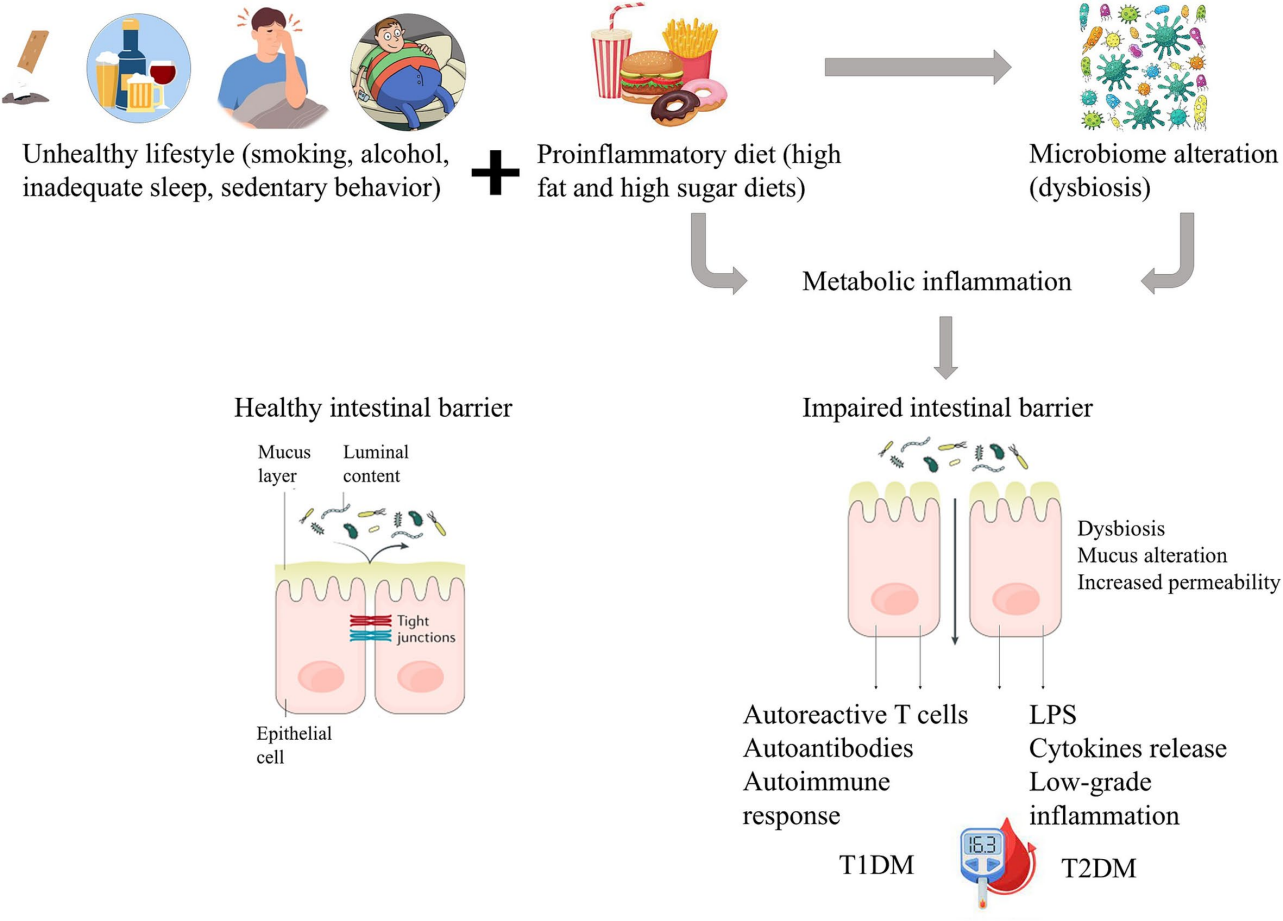
Schematic representation of the interplay between diet, gut microbiome, and intestinal barrier dysfunction in diabetes. In TD1M the increased gut permeability triggers an autoimmune response causing beta cell damage. In TD2M dysbiosis leads to low-grade inflammation and a reduction in insulin sensitivity.

Type 2 diabetes mellitus (T2DM) is a complex chronic metabolic disorder, that differs in many aspects from T1DM. Dysregulation of carbohydrate, lipid, and protein metabolism, typical for T2DM, is followed by insulin resistance or impaired insulin secretion, or a combination of both (DeFronzo et al., 2015). The risk factors for T2DM appear to be clearer than for T1DM and are closely related to environmental factors such as obesity and unwholesome lifestyle characterized by an unhealthy diet and physical inactivity. Although other factors such as mental stress, infections, and genetic predisposition should also be considered, obesity appears to be the most important factor contributing to the pathophysiological disturbances responsible for impaired glucose homeostasis. Obesity, characterized by excessive fat accumulation and low grade systemic and chronic inflammation, is closely related to the composition of the intestinal microbiota, and several works have highlighted a crucial role of the gut microbiota in the development of T2DM (Lambeth et al., 2015; Tai et al., 2015; Thingholm et al., 2019; Sikalidis and Maykish, 2020). The effect of inflammation on intestinal bacteria has not been yet fully understood, but systemic inflammation negatively affects the barrier function and immune system due to the action of pro-inflammatory cytokines, and consequently affects the composition and diversity of the microbiota (Riedel et al., 2022). Impaired gut barrier function can even result in metabolic endotoxemia (Sharma and Tripathi, 2019; Mohammad and Thiemermann, 2021). This adverse state is characterized by a diet-induced increase of bacterial lipopolysaccharide (LPS) levels in plasma, which leads to low-grade inflammation contributing to the development of a metabolic disease phenotype (Cani et al., 2007). Figure 1 presents the interplay between diet, gut microbiome and gut barrier dysfunction in diabetes mellitus.

Gut bacterial diversity and the number of butyrate-producing bacteria are significantly lower in patients with T2DM compared with healthy individuals (Qin et al., 2012; Tilg and Moschen, 2014; Candela et al., 2016; Zhao et al., 2019; Gurung et al., 2020), consistent with the same trend seen in type 1 diabetes. However, the results at the different taxonomic levels are quite contradictory, as described below.

Within Firmicutes, Clostridia was found to be dominant, however Larsen et al. (2010) found that this class was significantly lower in adult TD2 patients, whereas Zhang et al. (2013) described the opposite. The higher abundance of non-butyrate producing and opportunistic pathogenic Clostridiales was also observed by Karlsson et al. (2013) and Tilg and Moschen (2014), respectively, while according to Doumatey et al. (2020) the *Clostridiaceae* and *Peptostreptococcaceae* families (both class Clostridia) were lower in T2DM subjects. Within Proteobacteria, the class Betaproteobacteria was greatly increased in TD2 patients (Larsen et al., 2010; Zhang et al., 2013).

At the genus level, T2DM subjects were enriched with *Prevotella* (Zhang et al., 2013; Doumatey et al., 2020), *Dorea* (Zhang et al., 2013), *Subdoligranulum* (Qin et al., 2012; Zhang et al., 2013), *Ruminococcus* (Zhang et al., 2013; Candela et al., 2016), *Eubacterium* (Zhang et al., 2013; Zhao et al., 2019; Doumatey et al., 2020), *Sporobacter* (Zhang et al., 2013), *Abiotrophia* (Zhang et al., 2013), *Peptostreptococcus* (Zhang et al., 2013; Doumatey et al., 2020), *Collinsella* (Zhang et al., 2013; Lambeth et al., 2015; Candela et al., 2016), and *Lactobacilus* (Sato et al., 2014; Sedighi et al., 2017). Increased levels of *Coprococcus, Blautia*, the *Eubacterium hallii group*, and *Parasutterella* were described in T2DM cases by Zhao et al. (2019). However, these results are not consistently found in all published works. Opposite findings, i.e., significantly decreased abundances, were reported in T2DM patients for *Subdoligranulum* (Forslund et al., 2015; Zhang et al., 2019; Cunningham et al., 2021), *Prevotella* (Zhao et al., 2019), *Eubacterium* (Qin et al., 2012), *Ruminococcus* (Zhang et al., 2019), or *Collinsella* (Doumatey et al., 2020). Larsen et al. (2010) did not observe significant changes in relative abundances at the bacterial genus level.

At the species level, the increase of *Bacteroides caccae*, *Clostridium hathewayi*, *Clostridium ramosum*, *Clostridium symbiosum*, *Eggerthella lenta,* and *Escherichia coli* was described by Qin et al. (2012) in T2DM patients based on a metagenome-wide association study in Chinese cohort. Wu et al. (2020) described highly significant shifts (*p* < 0.001, mostly a decrease) in the abundance of 27 bacterial species in T2DM patients. Karlsson et al. (2013) described increased abundance of four *Lactobacillus* species and decreased abundance of five *Clostridium* species in the T2DM group of European women. The shifts in the microbiome in T2DM patients are presented in Supplementary Tables S1, S2. The inconsistency of the results can be caused by several parameters, in our opinion the composition of the study group is decisive, especially the age, gender, nationality, diet and lifestyle of the included individuals. However, assessing this problem is complicated as it can be influenced by a number of factors.

While the above mentioned genera and species are considered to be positively associated with T2DM, there is a group of beneficial gut bacteria that may play an important role in preventing and possibly treating the diabetes disease. Most studies have described low numbers of butyrate-producing bacteria such as *Eubacterium rectale*, *Faecalibacterium prausnitzii*, *Roseburia* sp. in T2DM patients, which were enriched only in healthy controls (Furet et al., 2010; Qin et al., 2012; Karlsson et al., 2013; Zhang et al., 2013, 2019; Forslund et al., 2015; Wu et al., 2020; Kwan et al., 2022). Butyrate is known to affect insulin sensitivity (Vrieze et al., 2012; Sanna et al., 2019), therefore, increased butyrate synthesis caused by an enhancement of the butyrate-producing intestinal bacteria is considered as one of the methods to prevent or treat diabetes (Zhang et al., 2021). On the other hand, butyrate can promote postprandial insulin secretion and propionate formation in the stool, which increases the risk of T2DM (Sanna et al., 2019; Zhang et al., 2021). Therefore, the role of propionate in T2DM will require more attention. In general, propionate is considered to be beneficial for health (Louis and Flint, 2017) showing positive effects on *β*-cell function, leading to increased insulin secretion (Pingitore et al., 2017). However, Sanna et al. (2019) described that abnormalities in propionate production or absorption are causally associated with increased T2DM risk.

*Bifidobacterium* is also considered a bacterium potentially protective against T2DM. This genus is consistently decreased in diabetics (Candela et al., 2016; Sedighi et al., 2017; Zhao et al., 2019; Ejtahed et al., 2020; Wu et al., 2020; He et al., 2023). It is well established that *Bifidobacteria* provide positive health benefits to their host through their metabolic activities by providing nutrients by degrading indigestible carbohydrates from the diet (Derrien et al., 2022), protecting against pathogens through competitive exclusion, reducing and treating gastrointestinal infections (O’Callaghan and van Sinderen, 2016), and eliciting various immune effects (e.g., decrease of pro-inflammatory cytokines such as TNF-*α*, IFN-*γ*, IL-2, IL-6, IL-17, IL-22, and IL-12; increase the number of CD4+ T cells, and IgA plasma cells) in the host (Dong et al., 2022). The effect of *Bifidobacteria* is intensively studied (Qian et al., 2022), but there is still a lack of knowledge about the molecular mechanisms explaining probiotic properties of *Bifidobacterium* strains.

*Akkermansia muciniphila* is thought to be negatively associated with T2DM, its decreased abundance in diabetic patients has been reported by several authors (Vaarala, 2013; Tilg and Moschen, 2014; Zhao et al., 2019; Gurung et al., 2020). *A. muciniphila* is an important bacterium that colonizes the human intestinal mucosa. Its metabolic activity is related to the integrity of the mucus layer, increasing mucus thickness, improving intestinal barrier function, and eliciting immune responses (Ottman et al., 2017). Studies in rodents showed a positive effect of *A. muciniphila* on host glucose metabolism (Everard et al., 2013; Greer et al., 2016). These findings induced the idea that *A. muciniphila* could be considered a promising next-generation probiotic (Ottman et al., 2017; Ropot et al., 2020). Several works however described elevated levels of this bacterium in T2DM cases (Qin et al., 2012; Zhang et al., 2013, 2021), suggesting that the results are inconclusive. Cekanaviciute et al. (2017) even described increased abundance of *A. muciniphila* in multiple sclerosis patients, raising the question of whether *A. muciniphila* is a beneficial bacterium with exclusively positive effects. Together with the above results, the possible use of *A. muciniphila* as a probiotic agent in diabetic patients should be supported by further thorough research.

*Faecalibacterium prausnitzii* is considered a beneficial bacterium providing positive health benefits. Its reduction is associated with various inflammatory diseases and several studies have reported a decrease in *F. prausnitzii* abundance in individuals with T2DM (Furet et al., 2010; Qin et al., 2012; Karlsson et al., 2013; Kwan et al., 2022). This species is crucial for the maintenance of gut homeostasis by producing short-chain fatty acids (SCFAs), which support gut epithelial integrity and contribute to protection against systemic inflammatory diseases (Effendi et al., 2022). The production of butyrate by *F. prausnitzii* is especially important, as it has anti-inflammatory effects, improves insulin sensitivity, and maintains the integrity of the intestinal epithelium (Maioli et al., 2021). Supplementation with *F. prausnitzii* has been associated with an improvement in gut barrier function and a reduction in inflammation, suggesting its potential as a therapeutic probiotic strain (He et al., 2021).

The metabolomic profile in T2DM, which is primarily driven by insulin resistance, shows clear changes compared to healthy controls, especially elevated levels of branched-chain amino acids (BCAAs), aromatic amino acids, and changes in organic acids (Roberts et al., 2014). Particularly, increased valine, maltose, glutamate, urate, and decreased glucuronolactone, lysine and lactate have been reported (Zhang et al., 2014). Unlike T1DM, lipid profile changes are less explored, but include higher concentrations of low-carbon lipids, such as glycerophospholipids. Organic acids and certain purine and urea cycle metabolites, such as arginine and citrulline, are also elevated in T2DM patients (Arneth et al., 2019).

Regarding gut permeability in T2DM patients, the impaired intestinal barrier function is associated rather to metabolic factors than autoimmunity (Di Vincenzo et al., 2024). Poor glycemic control weakens barrier integrity, increases inflammatory responses, and potentially exacerbates insulin resistance (Yuan et al., 2021). T2DM patients often have elevated levels of serum lipopolysaccharides (LPS), zonulin, and intestinal fatty acid-binding protein (IFABP), indicating compromised barrier function. This impairment contributes to chronic inflammation by allowing bacterial endotoxins to enter the bloodstream, which can exacerbate insulin resistance (Yuan et al., 2021).

The prevalence of T2DM is rising worldwide, and statistical data indicate a global epidemiology with no signs of stabilization. Similar to T1DM, type 2 diabetes is more widespread in U.S.A (11.6%) and developed regions of Western Europe, particularly in the Netherlands, Switzerland, and Sweden, where it exceeds 10%. Alarming is also the number of prediabetics and undiagnosed individuals whose world number is estimated at 30 and 44%, respectively (International Diabetes Federation, 2021). There are no gender differences in T2DM prevalence; men have a slightly higher incidence than women without being statistically significant, and men tend to be diagnosed at a younger age (Kautzky-Willer et al., 2023). Age is an important factor, as T2DM occurs in adults affecting about 15% of people at the age 50–70 years, 22% of the population over 70 years, but only 4.4% of younger people (15–49 years old) (Khan et al., 2020). These factors are linked to diet and lifestyle, and all of these aspects are related to the composition of the gut microbiome. Research focused on comparing different groups, whether by age, nationality, gender, medication, or health status, could elucidate specific changes in the microbiome and help understand the role of intestinal bacteria in T2DM disease in more specific contexts, with a greater opportunity to favorably influence gut microbiome composition.

## 6 Antidiabetic drugs and gut microbiome

As described above, in T1DM patients the beta cells in the pancreas are destroyed by the immune system and can no longer produce insulin. In T2DM patients, insulin is still produced, but the body is unable to use it properly due to insulin resistance. These characteristics are therefore reflected in a different treatment approach. People with type 1 diabetes must be treated by insulin (through multiple daily injections or an insulin pump), whereas in type 2 diabetes, blood glucose levels are lowered, insulin sensitivity and insulin secretion is improved through oral medication, with metformin generally being the first choice. However, several other classes of peroral glucose-lowering drugs, including selective inhibitor of sodium-glucose cotransporter 2 (SGLT2i), glucagon-like peptide 1 (GLP-1) receptor agonists, inhibitors of dipeptidyl peptidase 4 (DPP-4 inhibitors or gliptins), insulin sensitizer thiazolidinediones (glitazones), and insulin secretagogue sulfonylureas, may be used (Yang T. et al., 2023).

Antidiabetic drugs are directly or indirectly influenced by the composition of the gut microbiota, which may affect the therapeutic efficacy of treatment (Crudele et al., 2023). To assess the effect of insulin on intestinal bacteria, it would be necessary to compare the same patient group before treatment and at several time points after treatment, which would be very problematic to perform since patients with Type 1 DM are treated by insulin immediately from the beginning of their disease. Therefore, it is impossible to uncover the real impact of insulin on gut microbiota. However, studies show a negative correlation between HbA1c levels and the abundance of the family *Ruminococcaceae* (Mrozinska et al., 2021), the genus *Faecalibacterium* (Huang et al., 2018) and the genus *Akkermansia muciniphila* (Fassatoui et al., 2019) in T1DM patients, suggesting a positive effect of butyrate-producing bacteria.

Evaluating the effect of orally administered antidiabetic drugs is more straightforward because these medicinal substances pass through the digestive tract and interact with the gut microbiota. Intestinal bacteria can be sensitive to administered drugs or can chemically modified them and thus influence their pharmacological effect. The ability of human gut bacteria to metabolize orally administered drugs was documented by Zimmermann et al. (2019), who mapped 76 intestinal bacterial species and showed their metabolizing effect on two-thirds of 271 tested drugs (Zimmermann et al., 2019). Metformin, one of the most commonly prescribed drugs for T2DM patients, is known to have a strong impact on gut bacteria, as described in several review articles (Zhang and Hu, 2020; Hu et al., 2021; Kant et al., 2022; Ezzamouri et al., 2023).

Metformin is believed to influence the microbiome in the right direction, having a beneficial effect on lowering blood glucose levels and improving intestinal epithelial permeability and gut barrier function (Wu et al., 2017). This effect could be supported by increased abundances of the beneficial genera *Bifidobacterium, Faecalibacterium, Butyricicoccus, Butyrivibrio*, *Ruminococcus torques* and *Akkermansia muciniphila* (Forslund et al., 2015; De La Cuesta-Zuluaga et al., 2017; Lau et al., 2021). The positive effect of butyrate-producing bacteria is known and (Wu et al., 2017) observed a negative correlation between the increase in *Bifidobacterium adolescentis* and diabetes control (% of HbA1c), while any significant correlation was found between % of HbA1c and the abundance of *A. muciniphila*. Some other metformin-induced shifts, such as increased abundance of *Megasphaera* (De La Cuesta-Zuluaga et al., 2017; Wu et al., 2017) and *Turicibacter* (Zhang et al., 2019) or decreased abundance of *Intestinibacter bartlettii* (Wu et al., 2017; Mueller et al., 2021), could also be considered beneficial according to current knowledge. On the other hand, some authors detected the decrease in beneficial *Roseburia intestinalis, Roseburia faecis* (Hiel et al., 2020; Mueller et al., 2021) and *Oscillospira* (De La Cuesta-Zuluaga et al., 2017) and an increase in harmful *Salmonella, Klebsiella, Shigella* (Karlsson et al., 2013; Wu et al., 2017)*, Escherichia* (Karlsson et al., 2013; Forslund et al., 2015; De La Cuesta-Zuluaga et al., 2017; Wu et al., 2017; Hiel et al., 2020; Mueller et al., 2021) and *Fusobacterium* (Zhang et al., 2019). Assessing the impact of changes in some of the higher bacterial taxa is problematic, as individual species of families and genera can play very different roles in the gut microbiome. However, the metformin-associated microbiota seems to be more similar to healthy individuals than untreated diabetics (Crudele et al., 2023), and metformin is generally considered beneficial (Lv and Guo, 2020). Interestingly, the therapeutic effect of metformin is also influenced by the composition of the patient’s gut microbiota before starting the medication. The study of Elbere et al. (2020) showed an increased abundance of *Prevotella copri* at baseline in the group that did not respond to metformin treatment, while *Enterococcus faecium, Lactococcus lactis, Odoribacter* and *Dialister* were enriched in the responder group. In addition, *Streptococcus parasanguinis* was associated with the occurrence of severe side effects of metformin administration. Information on the state of the resident microbiota prior to treatment could therefore be useful to predict the tolerability of therapy. This is especially relevant in the context of personalized treatment, as differences in response to therapeutic interventions are often observed between individuals. The growing recognition that microbiome plays an important role in drug modification underscores the need to consider differential microbiota compositions and functions when developing treatment strategies. Future pharmaceutical developments should therefore consider how these variations may affect drug metabolism, absorption, efficacy, and toxicity. Additionally, understanding microbiota differences can help explain the different efficacies of generic drugs with similar active compounds (Zmora et al., 2016). The role of specific genera/species in the individual response to treatments should be taken into account and represents a new challenge in the study of the human microbiome.

Other oral antidiabetic agents are less researched and the results are inconsistent. Empagliflozin (SGLT2 inhibitor) significantly increased the richness and diversity of the gut microbiota, enriched the relative abundance of beneficial species of *Roseburia, Eubacterium* and *Faecalibacterium*, and decreased the harmful species of *Escherichia/Shigella, Bilophila* and *Hungatella* in the treatment-naïve T2DM patients (Deng et al., 2022). On the other hand, no changes in fecal bacteria composition were observed by van Bommel et al. (2020) when studying the effects of dapagliflozin. However, the T2DM participants in this study had been treated with metformin, which represents fundamentally different starting conditions. Liraglutide (GLP-1 agonist) significantly induced *Collinsella*, *Akkermansia* and *Clostridium* species and suppressed Fusobacteria (Shang et al., 2021), which is a positive result as some species of this phylum are associated with intestinal diseases such as inflammatory bowel disease (Lee et al., 2016) and colorectal cancer (Wang and Fang, 2023). In the study by Smits et al. (2021), liraglutide was administered to T2DM patients receiving stable doses of metformin and no effects on gut bacteria were observed. Similar results were obtained with sitagliptin (DPP-4 inhibitor) (Smits et al., 2021), indicating that the use of these drugs as add-on therapy to metformin is not associated with changes in the gut microbiota. This opinion is supported by the study of Wang Z. et al. (2021), who described a significant impact of both vildagliptin and saxagliptin on gut bacteria in T2DM patients not treated with metformin. After 3 months of treatment, vildagliptin decreased beneficial *Turicibacter* and increased *Megamonas*, which genus is thought to have a positive effect on human health (Yang X. et al., 2023). Saxagliptin reduced *Pseudomanas* and *Blautia* and increased beneficial *Faecalibacterium* and *Roseburia*, but also the opportunistic human pathogen *Klebsiella*. Acarbose (*α*-glucosidase inhibitor) inhibits not only human but also bacterial α-glucosidases, so its effects on the composition of the gut microbiome are to be expected. Increased abundance of *Bifidobacteria*, *Lactobacilli* (Su et al., 2015; Gu et al., 2017; Takewaki et al., 2021), *Eubacterium* (Takewaki et al., 2021) and *Enterococcus faecalis* (Su et al., 2015) and a reduction of *Bacteroides* (Gu et al., 2017; Takewaki et al., 2021) was associated with a decrease of inflammatory factors in plasma (Su et al., 2015). Overall, the results suggest a positive influence, however the effect of acarbose is highly dependent on diet and the original composition of the host gut microbiome (Takewaki et al., 2021). Only two clinical trials have investigated the impact of sulfonylureas on the gut microbiome in T2DM patients, but without evidence of positive effects (Gu et al., 2017; van Bommel et al., 2020). To date, no clinical study has been conducted to clarify the effect of treatment with thiazolidinediones on the human gut microbiota (Wang Z. et al., 2021). In summary, the antidiabetic drugs mainly enriched the beneficial *Faecalibacterium*, *Roseburia*, *Akkermansia muciniphila*, *Eubacterium* and *Bifidobacterium*, while the diminished taxa are the potentially pathogenic, such as *Escherichia*/*Shigella*, *Klebsiella* and *Fusobacterium*. However, this conclusion must be taken with caution, as studies vary in their conclusions, as noted above. Research in this area suggests that metformin and various other glucose-lowering agents, particularly SGLT2 inhibitors, DPP-4 inhibitors and α-glucosidase inhibitors, have a similar impact on the gut microbiota, primarily by promoting SCFA-producing bacteria and species with probiotic activity. Butyrate in particular is critical for insulin sensitivity and glycemic control. According to recent research by Martínez-López et al. (2024), metformin and linagliptin medications in combination with lifestyle changes also play an important role in people with prediabetes. The applied therapies improved insulin sensitivity, pancreatic *β*-cell function and increased the abundance of beneficial bacteria, such as *Roseburia*, *Bifidobacterium* and *Eubacterium hallii*, in prediabetics. Such an intervention could therefore reduce the incidence of T2DM. However, based on structural equation modeling, the authors concluded that the improvement in metabolic status in prediabetics was more related to pharmacological treatment, while bacterial changes had a minor impact. Further research is therefore needed to better understand the relationship between specific bacterial clusters and the regulation of insulin sensitivity, energy metabolism and systemic inflammation, and to uncover the complex interaction between the gut microbiota and the health status of the host.

## 7 Future challenges and research directions

**Figure 2:**
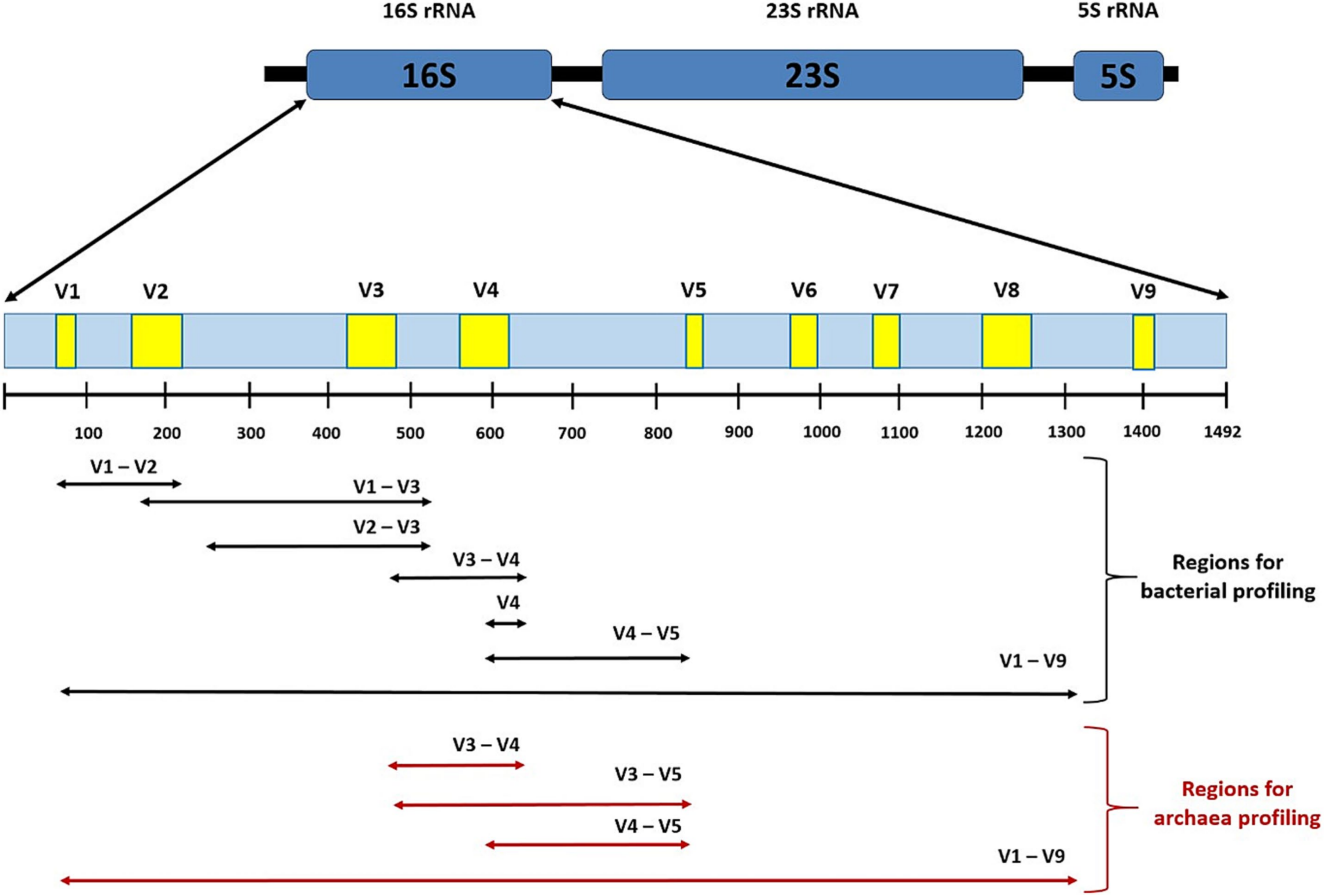
A schema of the prokaryotic rRNA operon with full-length 16S rRNA gene with variable regions V1-V9 showing the respective amplicons used for microbial profiling. For short-read-based NGS bacterial genotyping using Illumina or Ion Torrent device the primers targeting the following marked regions can be used: V1-V2, V1-V3 (Park et al., 2021; López-Aladid et al., 2023), V2-V3 (Bukin et al., 2019), V3-V4 (López-Aladid et al., 2023), hypervariable regions V4 alone (Bharti and Grimm, 2021), and V4-V5 (Fadeev et al., 2021). Regions V3-V4, V3-V5, and V4-V5 provide concurrent coverage of the archaeal domain (Pinto and Raskin, 2012; Takahashi et al., 2014; Fadeev et al., 2021), respectively. The V2 and V3 regions permit higher resolution at genera and species level (Bukin et al., 2019), while the V9 region provides the lowest resolution (Sirichoat et al., 2021). Long-read-based third generation sequencing targeting full V1-V9 regions using Sequel (PacBio technology) or MinION (Oxford Nanopor technology) device offers the best accuracy and discrimination between closely related species of bacteria (Matsuo et al., 2021; Park et al., 2021) and archaea (Meslier et al., 2022).

The amount of information supporting the important role of gut microbiota in relation to diabetes is steadily increased. The mainstream of research in this area focuses on next-generation sequencing (NGS) using bacterial 16S rRNA fragments (Figure 2). This marker mostly allows identification at the genus level, while identification at the species level is somewhat limited. For unidentified microorganisms, the NGS analysis provides part of results at the bacterial family or even order level. This deficiency makes it impossible to properly evaluate the results and identify beneficial or, on the contrary, harmful bacteria. An example of this is the genus *Ruminococcus*, which includes beneficial species such as *R. bromii* and on the other hand Crohn’s disease associated *R. gnavus* (Henke et al., 2019). By using the full 16S rRNA gene (~1,500 bp), the weakness of the method based on amplification of part of the variable region can be overcome and this approach becomes realistic (Johnson et al., 2019; Jeong et al., 2021). A major step forward is also the PacBio third-generation long-read sequencing platform, whose widespread use is hampered by the high cost of metagenomics analysis and the need for large amounts of samples (micrograms of DNA, while NGS requires nanograms of DNA).

**Figure 3:**
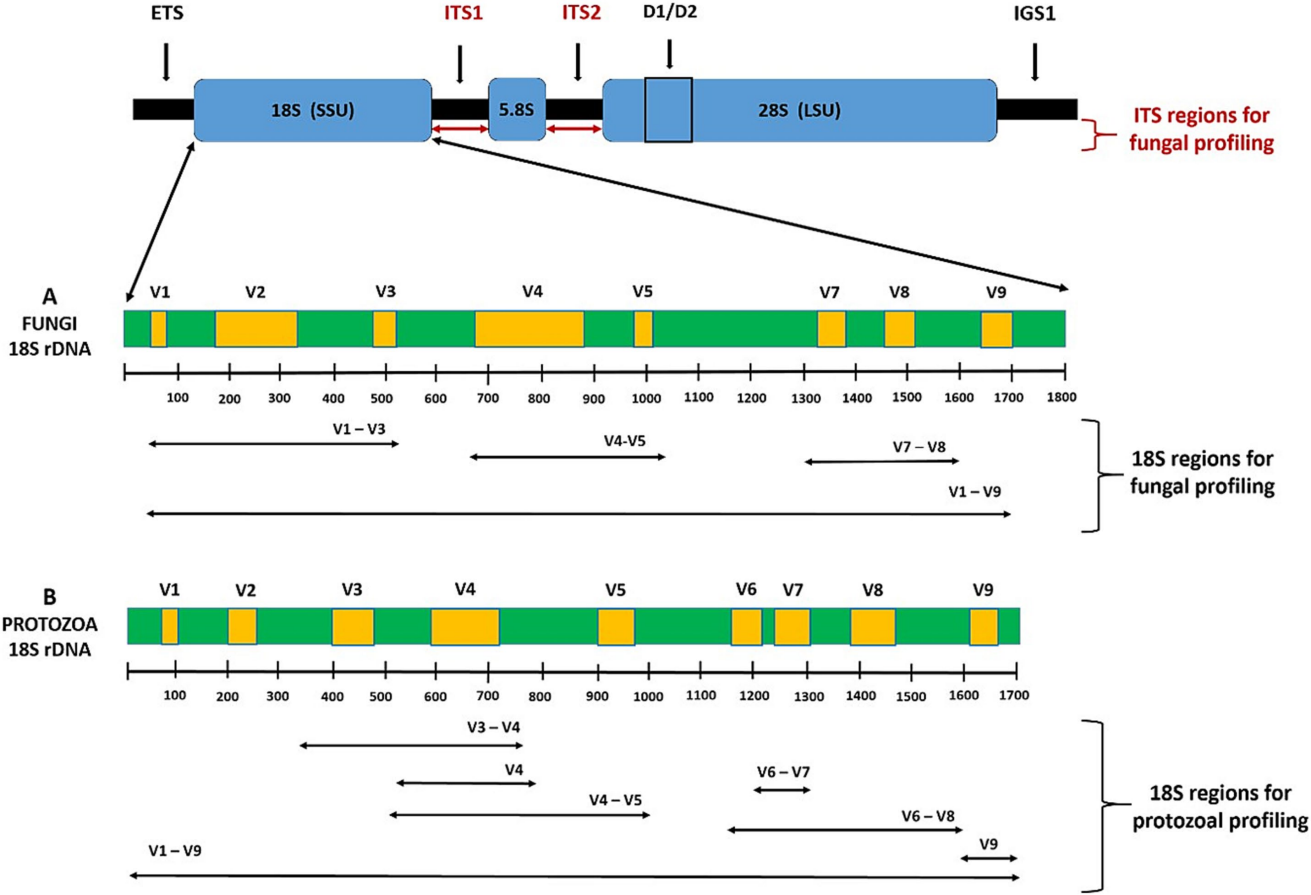
**(A-B)** A schema of the eukaryotic rDNA operon with full-length 18S rRNA gene with variable regions V1-V9 showing the respective amplicons used for microbial profiling. For NGS fungal genotyping using Illumina or Ion Torrent device the primers targeting the following marked 18S regions can be used: V1-V3 (Banos et al., 2018; Hoggard et al., 2018), V4-V5 (Wu et al., 2015; Banos et al., 2018), V7-V8 (Banos et al., 2018). Some regions are group specific, thus primers have to be selected according to fungi of interest (Banos et al., 2018). Long-read-based third generation sequencing targeting full V1-V9 regions using Sequel (PacBio technology) or MinION (Oxford Nanopor technology) device offers the best accuracy and discrimination between closely related species of fungi (D’Andreano et al., 2020). Fungal ITS1 and ITS2 (internal transcribed spacer) regions are more variable and suitable as the genetic marker to measure intraspecific genetic diversity (Schoch et al., 2012; Yang et al., 2018; Raimondi et al., 2019). 18S rRNA is mainly used for high resolution taxonomic studies of fungi, while the ITS region is mainly used for fungal diversity studies as a fungal barcode marker. The best results would be achieved with third generation sequencing of amplicons of the whole fungal ribosomal operon (i.e., ETS, 18S, ITS1, 5.8S, ITS2, 28S, and IGS), which is however expensive and demanding on bioinformatics (Wurzbacher et al., 2019). For NGS protozoal genotyping using Illumina or Ion Torrent device the primers targeting the following marked 18S regions can be used: V3-V4 (Ishaq and Wright, 2014), V4 (Maritz et al., 2019), V4-V5 (Maloney et al., 2019), V6-V7 (Bhute et al., 2017b), V6-V8 (Ishaq and Wright, 2014), and V9 (Maritz et al., 2019). Long-read-based third generation sequencing targeting the full V1-V9 protozoal regions using Sequel (PacBio technology) or MinION (Oxford Nanopor technology) device offers the best accuracy and species discrimination (Gad et al., 2023).

Another challenge lies in the study of other components of the microbiome. The bacterial 16S rRNA marker should be complemented by other markers covering methanogens (Figure 2), fungi, protozoa (Figure 3), and viruses to provide a more representative picture of microbial diversity and structure. Methanogens are a less numerous but important component of the human gastrointestinal tract. These microorganisms are involved in the final steps of fermentation processes in the human gut due to their syntrophic interactions with bacteria. Intestinal methanogens, mostly hydrogenotrophs, use H_2_ (and often formate) to reduce CO_2_ to methane. However, their diversity is still incomplete (Borrel et al., 2020), and their clinical relevance has not yet been clearly assessed. Methanogenic archaea have been largely overlooked in human studies, but a growing body of work points to their association with various diseases, including colorectal cancer (CRC), inflammatory bowel disease (IBD), irritable bowel syndrome (IBS), diverticulosis, chronic constipation, and obesity (Djemai et al., 2022; Hoegenauer et al., 2022). Microbiome studies in diabetic patients have mostly omitted methanogens, with only the study by Bhute et al. (2017b) describing an increase of *Methanobrevibacter* associated with a decrease in *Methanosphaera* genus in T2DM patients.

The role of fungi has also been neglected in diabetic patient studies, but recent research suggests a significant impact of intestinal mycobiota on host health (van Tilburg Bernardes et al., 2020). A limited body of work addressing this issue suggests higher diversity of species belonging to *Candida* genus in T1DM children, and interestingly, fungal species were more resistant to antifungal treatment in these patients (Kowalewska et al., 2016). The increased incidence of *Candida* spp. was also observed in adults with both types of diabetes (Gosiewski et al., 2014; Bhute et al., 2017b; Jayasudha et al., 2020), indicating a detrimental role of this genus in such diseases and gut bacterial microbiome assembly. According to Zhang et al. (2022), an increase in *Candida* spp. is consistently observed in urbanisation-associated diseases (obesity and inflammatory bowel disease), which we believe may include also diabetes.

Other gut microorganisms that have not yet been studied are protozoa. These unicellular eukaryotes were previously thought to be parasites that cause gastroenteritis problems. However, recent research indicates that *Blastocystis* species and *Dientamoeba fragilis* are more likely enteric commensals (Guzzo et al., 2022). They have been found to be the most abundant protozoa in the stool of healthy humans (de Boer et al., 2020) and have even been associated with increased bacterial diversity in the gut (Nash et al., 2017). The only mention of *Blastocystis* in diabetics was published by Bhute et al. (2017b), who found no differences in protozoan abundance between healthy controls and T2DM patients. However, the inclusion of eukaryotic organisms in microbial analysis would expand and improve the overview of the composition, diversity, and dysbiosis of the gut microbiome of diabetic patients.

The greatest challenge is probably the study of intestinal bacteriophages (phages), viruses that infect intestinal bacteria. Phages are simple organisms composed of nucleic acid (DNA or RNA, double- or single-stranded) packed in a protein capsid. They are parasitic by nature, requiring a bacterial host to reproduce. The phage inserts its genetic material into the bacterial cell, and this infection is followed by a lytic or lysogenic life cycle. Lytic phages use the bacterium to produce phage components, then destroy the cell and release new phage particles. Lysogenic phages incorporate their nucleic acid into the chromosome and replicate with the cell without destroying the host bacterium. In this way, phages can invade bacteria and thus transfer some additional genetic traits, such as antibiotic resistance genes. Thus, both types of cycles can affect the populations of intestinal bacteria and change their composition. In addition, phages interact with the host immune system, affecting the homeostasis of the GIT (Zuppi et al., 2022). The gut virome is an emerging topic in the study of the human microbiome, and several studies have indicated a link between the gut virome and various diseases (Bai et al., 2022), including T1DM (Zhao et al., 2017; Kim et al., 2019; Tetz et al., 2019; Vehik et al., 2019; Cinek et al., 2021) and T2DM (Ma et al., 2018; Chen et al., 2021). Although viruses are highly abundant in the gut and have significant impacts on microbial ecosystems, they are still the least studied members of the gut microbiome, and the genomic diversity of gut phages is still largely unknown (Zuppi et al., 2022).

All of these microorganisms can be identified simultaneously through shotgun sequencing. This approach, also called whole genome sequencing (WGS), enables the sequencing of the entire genome, i.e., not only rRNA, but identifies all genes (metagenome) and thus can provide additional information about the functional potential of the microbiome (Ranjan et al., 2016; Durazzi et al., 2021). WGS method is however more expensive and requires sequencing with high coverage and analysis with more extensive data.

Culture-independent research has brought great progress in understanding the composition and diversity of the gut microbiome in the context of disease. However, these methods have also revealed a large number of unknown, uncultured bacteria whose role in the digestive tract remains unknown. Therefore, great efforts should be directed toward the cultivation of intestinal microorganisms to determine the biochemical and metabolic properties of such bacterial species. Isolation of individual species is complicated by the difficulty of operating under strictly anaerobic conditions and the lack of knowledge about proper growth requirements and suitable substrates. A considerable amount of bacterial species thus still lack a cultured representative (Hitch et al., 2021). Several large projects in recent years focused on the cultivation of microbes from the human gut have contributed significantly to increasing the number of new bacterial strains available in public collections of microorganisms (Browne et al., 2016; Lagier et al., 2018; Forster et al., 2019; Zou et al., 2019). However, Clavel et al. (2022) pointed out that isolating and studying individual microorganisms is not sufficient because the functions of a strain depend heavily on its interaction with other members of the microbial community and its interaction with the host. One approach to study host–microbe interactions is based on defined synthetic bacterial communities representing the human gut microbiota that can be applied to gnotobiotic animals or to sophisticated *in vitro* cell models (Elzinga et al., 2019). Another approach is the so-called culturomics (Lagier et al., 2018). This is a high-throughput culture method that allows the identification of unknown bacteria inhabiting the human gut. The basis of the method lies in the combination of *in vitro* cultivation of stool samples with matrix assisted laser desorption ionization-time of flight mass spectrometry (MALDI-TOF MS) identification. This setup can use different selective culture conditions, detects only viable bacteria, and thus solves the problem of ‘non-cultivable’ or ‘non-cultured’ bacteria (Greub, 2012; Lagier et al., 2018).

The expansion of microbial markers and the application of culture methods in the study of the gut microbiome of diabetic patients will provide new insights and help clarify the extent to which the microbiota contributes to disease initiation and progression, and allow better application of therapeutic interventions such as probiotic administration, dietary fiber intervention, or fecal microbiota transplantation (FMT). FMT appears to be a promising approach to reconstruct the gut microbiota in both T1DM (De Groot et al., 2021; Xie et al., 2022) and T2DM patients (Aron-Wisnewsky et al., 2019; Wang et al., 2020; Su et al., 2022) resulting in improved insulin sensitivity, weight loss, reductions in fasting blood glucose and glycated hemoglobin, increased abundance of *Bifidobacterium* and reduction of pro-infammatory sulfate-reducing bacteria (*Bilophila*, *Desulfovibrio*). Detailed analysis of the intestinal microbiome is therefore urgently needed to avoid possible transmission of unwanted or even harmful microorganisms. Research in this field is very important because a reliable definition of the “healthy microbiome” is still lacking (Li D. et al., 2020; Najmanová et al., 2022).

Further challenges lie in gaining a deeper insight into endocrinology, immunology and metabolomics as well as in a more complex combination of these areas with the investigation of the microbiome of diabetics. The SCFAs produced by gut bacteria are involved in the indirect regulation of neuroactive compounds and hormones, and the gut microbiota is able to produce various substances of a hormonal nature. Investigating the novel microbial immunomodulatory metabolites and understanding their effect on host immunity and pancreatic functions thus represents a complicated study goal, but one that could yield significant progress. A better understanding of the interactions between drugs and the gut microbiome and exploring the effects of different therapeutic combinations on the intestinal bacteria is also a challenging topic. Continued research is needed to gain a more comprehensive understanding of the interplay between the gut ecosystem and diabetes and to provide a substantial basis for the prediction and treatment of diabetes in the future.

## 8 Conclusion

The gut microbiota of patients suffering from both types of diabetes mellitus is characterized by reduced bacterial diversity, a lower number of butyrate-producing bacteria, especially *Roseburia* and *Faecalibacterium*, and a low abundance of bacteria with probiotic activity, namely *Bifidobacterium* and *Akkermansia*. Most diabetes publications agree on an increased incidence of Bacteroidetes and a decrease in Firmicutes, but there is no consensus on specific or typical biomarkers. Studies should therefore be more personalized and a multidisciplinary and well-coordinated research approach would be useful to elucidate the functional and metabolic contribution of individual microbes and provide evidence-based data when considering the gut microorganisms as a putative target for the prevention of metabolic disorders. A growing body of literature confirms a link between the structural composition of the intestinal microbiota and diabetes. Despite intensive studies, it is still unclear whether the changes in the microbiome are causal or as a consequence of the disease process, and bacterial heterogeneity does not allow simple interpretations of the results. It is still questionable whether gut bacteria could play a direct role in the prevention and treatment of disease. However, scientists are striving to use the gut microorganisms not only as a biomarker for diabetes but also as a promising target for therapeutic interventions.

The significant increase in the number of diabetes cases is alarming and, in our opinion, the situation is so serious that this problem should be seen as a social problem and not just a health problem. A lifestyle characterized by stress, fast food and lack of exercise is a global problem and should not only be perceived as a health issue by the state administration. More effort should be put into prevention and education, proper dietary habits, incorporating adequate physical activity into everyday life and boosting immunity. Attention should be focused on new treatment options for diabetes, but also on non-pharmacological strategies, such as the use of gut biotics like prebiotics, probiotics and synbiotics. All these factors can modulate the gut microbiome, prevent gut dysbiosis and play a role in the treatment of diabetes.
