# Supplementary material for: Diabetes and gut microbiome
Source: Front Microbiol. 2025 Jan 7;15:1451054. doi: 10.3389/fmicb.2024.1451054 (PMC11747157; doi:10.3389/fmicb.2024.1451054)
Supplement: Supplementary file 1 [file Data_Sheet_1.DOCX]

Supplementary Material

**DIABETES AND GUT MICROBIOME**

**Kateřina Olša Fliegerová^1^, Tiziana Maria Mahayri^1,2^*, Hana Sechovcová^1,3^, Chahrazed Mekadim^1^, Jakub Mrázek^1^, Radka Jarošíková^4, 5^, Michal Dubský^4^, Vladimíra Fejfarová^4, 5^**

^1^ Institute of Animal Physiology and Genetics, CAS, Laboratory of Anaerobic Microbiology, Prague, Czech Republic

^2^ Department of Veterinary Medicine, University of Sassari, 07100 Sassari, Italy

^3^ Czech University of Life Sciences, Faculty of Agrobiology, Food and Natural Resources, Department of Microbiology, Nutrition and Dietetics, Prague, Czech Republic

^4^ Diabetes Centre, Institute for Clinical and Experimental Medicine, Prague, Czech Republic

^5^ Department of Internal Medicine, Second Faculty of Medicine, Charles University, Prague, Czech Republic

**Table S1. Consistent gut microbiota changes in patients with type 1 and type 2 diabetes mellitus**

| **Bacteria** | **T1DM** | **T2DM** | **References** |
| --- | --- | --- | --- |
| Bacteroidetes | Increased |  | (Giongo et al. 2011; Brown et al. 2011; Vaarala 2012; De Goffau et al. 2013; Qi et al. 2016) |
| Firmicutes | Decreased |  | (Giongo et al. 2011; Brown et al. 2011; Vaarala 2012; De Goffau et al. 2013; Qi et al. 2016) |
| Verrucomicrobiae |  | Decreased | (Zhang et al. 2013) |
| Proteobacteria |  | Increased | (Zhao et al. 2019) |
| Betaproteobacteria |  | Increased | (Larsen et al. 2010; Zhang et al. 2013) |
| *Bacteroidaceae* | Increased |  | (De Goffau et al. 2013; Leiva-Gea et al. 2018) |
| *Rikenellaceae* | Increased |  | (Giongo et al. 2011; Leiva-Gea et al. 2018) |
| *Prevotellaceae* | Increased |  | (Leiva-Gea et al. 2018) |
| *Streptococcaceae* | Increased |  | (Leiva-Gea et al. 2018) |
| *Enterobacteriaceae* | Increased |  | (Soyucen et al. 2014; Leiva-Gea et al. 2018) |
| *Lachnospiraceae* | Decreased |  | (Giongo et al. 2011; Leiva-Gea et al. 2018) |
| *Bifidobacteriaceae* | Decreased |  | (Leiva-Gea et al. 2018) |
| *Eubacteriaceae* | Decreased |  | (Giongo et al. 2011) |
| *Bacteroides* |  |  | (Giongo et al. 2011; Brown et al. 2011; De Goffau et al. 2013; Leiva-Gea et al. 2018) |
| *Blautia* | Increased | Increased | (Qi et al. 2016; Leiva-Gea et al. 2018) |
| *Veillonella* | Increased |  | (Brown et al. 2011; Leiva-Gea et al. 2018) |
| *Streptococcus* | Increased |  | (Brown et al. 2011; De Goffau et al. 2014; Leiva-Gea et al. 2018) |
| *Clostridium* | Increased |  | (Giongo et al. 2011; De Goffau et al. 2013) |
| *Sutterella* | Increased |  | (Leiva-Gea et al. 2018) |
| *Enterobacter* | Decreased |  | (Leiva-Gea et al. 2018) |
| *Alistipes* | Decreased |  | (Brown et al. 2011) |
| *Ruminococcus* | Decreased |  | (Giongo et al. 2011; De Goffau et al. 2014; Leiva-Gea et al. 2018) |
| *Lachnospira* | Decreased |  | (Qi et al. 2016; Leiva-Gea et al. 2018) |
| *Roseburia* | Decreased |  | (Brown et al. 2011; De Goffau et al. 2013; Cinek et al. 2018; Leiva-Gea et al. 2018) |
| *Anaerostipes* | Decreased |  | (Brown et al. 2011; Leiva-Gea et al. 2018) |
| *Faecalibacterium* | Decreased |  | (Giongo et al. 2011; Brown et al. 2011; Huang et al. 2018; Leiva-Gea et al. 2018) |
| *Eubacterium* | Decreased |  | (Giongo et al. 2011; Brown et al. 2011; Cinek et al. 2018) |
| *Bifidobacterium* |  | Decreased | (Candela et al. 2016; Sedighi et al. 2017; Zhao et al. 2019; Ejtahed et al. 2020; Wu et al. 2020; He et al. 2023) |
| *Akkermansia* | Decreased |  | (Brown et al. 2011) |
| *Lactobacillus* | Decreased |  | (De Goffau et al. 2014; Alkanani et al. 2015) |
| *Escherichia* | Increased |  | (Cinek et al. 2018) |
| *Dorea* |  | Increased | (Zhang et al. 2013) |
| *Sporobacter* |  | Increased | (Zhang et al. 2013) |
| *Abiotrophia* |  | Increased | (Zhang et al. 2013) |
| *Peptostreptococcus* |  | Increased | (Zhang et al. 2013; Doumatey et al. 2020) |
| *Lactobacilus* |  | Increased | (Sato et al. 2014; Sedighi et al. 2017) |
| *Coprococcus* |  | Increased | (Zhao et al. 2019) |
| *Eubacterium hallii group* |  | Increased | (Zhao et al. 2019) |
| *Bacteroides caccae* |  | Increased | (Qin et al. 2012) |
| *Clostridium hathewayi* |  | Increased | (Qin et al. 2012) |
| *Clostridium ramosum* |  | Increased | (Qin et al. 2012) |
| *Clostridium symbiosum* |  | Increased | (Qin et al. 2012) |
| *Eggerthella lenta* |  | Increased | (Qin et al. 2012) |
| *Escherichia coli* |  | Increased | (Qin et al. 2012) |
| *Lactobacillus* species |  | Increased | (Karlsson et al. 2013) |
| *Clostridium* species |  | Decreased | (Karlsson et al. 2013) |
| *Eubacterium rectale* |  | Decreased | (Qin et al. 2012; Karlsson et al. 2013; Zhang et al. 2013; Wu et al. 2020) |
| *Faecalibacterium prausnitzii* |  | Decreased | (Furet et al. 2010; Qin et al. 2012; Karlsson et al. 2013; Zhang et al. 2013, 2019; Wu et al. 2020; Kwan et al. 2022) |
| *Roseburia* sp |  | Decreased | (Qin et al. 2012; Karlsson et al. 2013; Zhang et al. 2013; Forslund et al. 2015) |
| *Akkermansia muciniphila* |  | Decrease | (Vaarala 2013; Tilg and Moschen 2014; Zhao et al. 2019; Gurung et al. 2020) |

**Table S2. Contradictory gut microbiota changes in patients with type 1 and type 2 diabetes mellitus**

| **Bacteria** | **T1DM** | **T2DM** | **References** |
| --- | --- | --- | --- |
| Firmicutes |  | Increased | (Zhao et al. 2019) |
|  |  | Decreased | (Larsen et al. 2010; Doumatey et al. 2020) |
| Bacteroidetes |  | Increased | (Doumatey et al. 2020) |
|  |  | Decreased | (Zhao et al. 2019) |
| Actinobacteria | Increased |  | (Brown et al. 2011) |
|  | Decreased |  | (Leiva-Gea et al. 2018) |
| Proteobacteria | Increased |  | (Brown et al. 2011) |
|  | Decreased |  | (Leiva-Gea et al. 2018) |
| Clostridia |  | Increased | (Zhang et al. 2013) |
|  |  | Decreased | (Larsen et al. 2010) |
| *Clostridiaceae* |  | Increased | (Karlsson et al. 2013; Tilg and Moschen 2014) |
|  |  | Decreased | (Doumatey et al. 2020) |
| *Peptostreptococcaceae* |  | Increased | (Karlsson et al. 2013; Tilg and Moschen 2014) |
|  |  | Decreased | (Doumatey et al. 2020) |
| *Ruminococcaceae* | Increased |  | (Leiva-Gea et al. 2018) |
|  | Decreased |  | (Huang et al. 2018) |
| *Veillonellaceae* | Increased |  | (Giongo et al. 2011; Leiva-Gea et al. 2018) |
|  | Decreased |  | (Huang et al. 2018) |
| *Prevotella* | Increased | Increased | (Zhang et al. 2013; Alkanani et al. 2015; Leiva-Gea et al. 2018; Doumatey et al. 2020) |
|  | Decreased | Decreased | (Brown et al. 2011; Zhao et al. 2019) |
| *Lactoabacillus* | Increased |  | (Brown et al. 2011) |
|  | Decreased |  | (Alkanani et al. 2015; Leiva-Gea et al. 2018) |
| *Lactococcus* | Increased |  | (Brown et al. 2011) |
|  | Decreased |  | (Alkanani et al. 2015; Leiva-Gea et al. 2018) |
| *Bifidobacterium* | Increased |  | (Brown et al. 2011) |
|  | Decreased |  | (Alkanani et al. 2015; Leiva-Gea et al. 2018) |
| *Subdoligranulum* |  | Increased | (Qin et al. 2012; Zhang et al. 2013) |
|  |  | Decreased | (Forslund et al. 2015; Zhang et al. 2019; Cunningham et al. 2021) |
| *Eubacterium* |  | Increased | (Zhang et al. 2013; Zhao et al. 2019; Doumatey et al. 2020) |
|  |  | Decreased | (Qin et al. 2012) |
| *Ruminococcus* |  | Increased | (Zhang et al. 2013; Candela et al. 2016) |
|  |  | Decreased | (Zhang et al. 2019) |
| *Collinsella* |  | Increased | (Zhang et al. 2013; Lambeth et al. 2015; Candela et al. 2016) |
|  |  | Decreased | (Doumatey et al. 2020) |
